# Functionalization of PET Track-Etched Membranes by UV-Induced Graft (co)Polymerization for Detection of Heavy Metal Ions in Water

**DOI:** 10.3390/polym11111876

**Published:** 2019-11-13

**Authors:** Maxim V. Zdorovets, Ilya V. Korolkov, Arman B. Yeszhanov, Yevgeniy G. Gorin

**Affiliations:** 1 L.N.Gumilyov Eurasian National University, Satpaev str., 5, Nur-Sultan 010008, Kazakhstan; arman_e7@mail.ru (A.B.Y.); gorineg@mail.ru (Y.G.G.); 2 The Institute of Nuclear Physics, Ibragimov str., 1, Almaty 050032, Kazakhstan; 3 Ural Federal University, Mira str. 19, Ekaterinburg 620002, Russia

**Keywords:** track-etched membranes, graft polymerization, stripping voltammetry, 4-vinylpyridine, acrylic acid

## Abstract

Nowadays, water quality monitoring is an essential task since environmental contamination and human exposure to heavy metals increased. Sensors that are able to detect ever lower concentrations of heavy metal ions with greater accuracy and speed are needed to effectively monitor water quality and prevent poisoning. This article shows studies of the modification of flexible track-etched membranes as the basis for the sensor with various polymers and their influence on the accuracy of detection of copper, cadmium, and lead ions in water. We report the UV-induced graft (co)polymerization of acrylic acid (AA) and 4-vinylpyridine (4-VPy) on poly(ethylene terephthalate) track-etched membrane (PET TeMs) and use them after platinum layer sputtering in square wave anodic stripping voltammetry (SW-ASV) for detection of Cu^2+^, Cd^2+^, and Pb^2+^. Optimal conditions leading to functionalization of the surface and retention of the pore structure were found. Modified membranes were characterized by SEM, FTIR, X-ray photoelectron spectroscopy (XPS) and colorimetric analysis. The dependence of the modification method on the sensitivity of the sensor was shown. Membrane modified with polyacrylic acid (PET TeMs-g-PAA), poly(4-vinylpyridine) (PET TeMs-g-P4VPy), and their copolymer (PET TeMs-g-P4VPy/PAA) with average grafting yield of 3% have been found to be sensitive to µg/L concentration of copper, lead, and cadmium ions. Limits of detection (LOD) for sensors based on PET TeMs-g-PAA are 2.22, 1.05, and 2.53 µg/L for Cu^2+^, Pb^2+^, and Cd^2+^, respectively. LODs for sensors based on PET TeMs-g-P4VPy are 5.23 µg/L (Cu^2+^), 1.78 µg/L (Pb^2+^), and 3.64 µg/L (Cd^2+^) µg/L. PET TeMs-g-P4VPy/PAA electrodes are found to be sensitive with LODs of 0.74 µg/L(Cu^2+^), 1.13 µg/L (Pb^2+^), and 2.07 µg/L(Cd^2+^). Thus, it was shown that the modification of membranes by copolymers with carboxylic and amino groups leads to more accurate detection of heavy metal ions, associated with the formation of more stable complexes.

## 1. Introduction

Currently, development of analytical methods for detection of heavy metal ions is urgent task since toxic ions such as As, Pb, Cd, Hg, Ni, and others are widely used in industry and released into the environment affecting human health [1,2]. Accumulation of heavy metal ions in the body can cause different diseases such as cancer, schizophrenia; kidney, lung, liver, skin diseases, etc. The World Health Organization has established the maximum permissible concentration of toxic metals in water, which, for instance, for Pb^2+^ is 0.01 mg/L [3]. Methods of absorption spectroscopy, mass spectrometry, and X-ray fluorescence spectroscopy can be used to detect metal ions in trace level [4]. However, these methods have drawbacks including bulkiness, high cost, time-consuming, etc. In this regard, interest is growing in the search for portable, inexpensive, and sensitive methods of heavy metal ion analysis. One such method is electrochemical method based on stripping voltammetry [5]. This method is currently widely used both in the field of chemical analysis of substances present in trace-level concentrations [6,7], and to study the mechanism of electrode reactions, the properties of solid electrodes, the adsorption of substances, etc.; it is inexpensive, fast, and portable. Moreover, advantages of the method are: the ability to determine a significant number (more than 40) of chemical elements and many organic compounds; low detection limits (10^−9^–10^−10^M); high selectivity and good metrological characteristics; ease of computerization and automation.

The stripping voltammetry is usually performed with a three-electrode system containing a reference electrode, auxiliary electrode, and working electrode (WE). Electrodes based on mercury and bismuth are the most commonly used for standard tests. Mercury and bismuth are toxic and difficult to use, and new materials are currently under considerable attention [8]. For such purpose, materials based on carbon nanotubes [9], carbon thin film [10], graphene and graphene oxide [11], metal nanoparticles [11,12,13,14], and polymers and membranes [15,16,17] are used as WE. Moreover, the number of publications has been growing on the use of track-etched membranes (TeMs) based on polyvinylidene fluoride (PVDF) as a dual-electrode [18,19,20,21,22,23]. TeMs are thin (5–24 µm), light, and flexible polymeric films with pores with narrow pore-size distribution. It is possible to control the pore size from 30–50 nm to 3–6 microns, as well as the shape of the channel, which can be cylindrical, tapered, and others [24,25], subjected to relatively easy modification by functional polymers, which makes them an attractive object for research in voltammetric measurements as WE. To increase the selectivity and sensitivity of the analysis, modification of the TeMs surface with functional monomers is carried out.

Barsbay et al. [20] grafted poly(acrylic acid) (PAA) into the nanochannels of ß-PVDF TeMs by controlled radical polymerization using reversible addition-fragmentation chain transfer (RAFT) agents. Such grafted membranes were transformed into membrane electrode by deposition of gold layer (50 nm) and found to be sensitive to sub-ppb Pb^2+^ concentration. Poly(4-vinyl pyridine) (P4VPy) grafted into PVDF was found to be effective in detection of mercury [19,26] with concentrations ng/L after 24 h of absorption and µg/L after 2 h of absorption. Penaeva et al. [18] reported the electron-beam-induced graft polymerization of bis[2-(methacryloyloxy)ethyl] phosphate onto PVDF membrane for the detection of uranium (VI) in ppb concentrations from 20 to 100 ppb using square wave cathodic stripping voltammetry.

At the same time, there are no works in the literature on the modifications of TeMs for stripping voltammetry by copolymers with different nature. However, it is known that such copolymers form more stable complexes with heavy metal ions [27,28], which ultimately will improve the properties of the sensor [29]. Such polymers can be PAA and P4VPy. PAA [20,30] and P4VPy [19,31] were found to be effective modifier of electrodes for the detection of lead, cadmium, cobalt, and others. Their copolymers can form more stable complexes with heavy metal ions [32,33]. Thus, using PAA-P4VPy copolymers as modifying agents can improve the versatility, sensitivity as well as enable its use in various electrochemical applications at a much larger scale.

The current study is focused on investigating the effect of copolymer with carboxylic and amino groups or individual polymers on sensitivity of heavy metal ion electrochemical detection. Modification of TeMs based on PET was performed by simple method of UV-induced graft polymerization of acrylic acid (AA) and 4-vinylpyridine (4VPy) and their copolymers. Based on such modified membranes, sensors were prepared by platinum sputtering. These sensors were tested for detection of copper, lead, and cadmium ions in the concentration range from 0.25 to 12.5 µg/L by the method of stripping voltammetry.

## 2. Materials and Methods

### 2.1. Materials and Instruments

Acrylic acid, 4-vinylpyrridine, benzophenone, *N*,*N*-dimethylformamide, and ethanol (reagent grade) were purchased from Sigma-Aldrich. Acrylic acid was purified by distillation, 4-vinylpyrridine was purified by Al2O3 column chromatography, and benzophenone was cleaned by recrystallization.

Sodium acetate, lead, cadmium, and copper standard solutions (Sigma-Aldrich, Hong Kong, China) were analytical grade and used without further purification. Deionized water purchased from Akvilon-D301 (18.2 MΩ) was used for preparing all the solutions. A PalmSens EmStat 3+ potentiostat (Houten, The Netherlands) was used for voltammetric measurements.

### 2.2. Preparation of the Membranes and Their Modification

TeMs were prepared by irradiation of 12 µm thick PET films with krypton ions with an energy of 1.75 MeV/nucleon and ion fluence of 4.3·10^7^ ion/cm^2^ using the DC-60 accelerator in Institute of Nuclear Physics (Nur-Sultan, Kazakhstan). Membranes with average pore diameters of 400 nm were obtained by photosensitization and chemical etching in 2.2 M NaOH at 85 °C.

Modification of poly(ethylene terephthalate) track-etched membranes (PET TeMs) was performed by photoinduced graft (co)polymerization of AA and 4-VPy. The samples were ultrasonicated for 10 min (in water) to remove any pollutions and then soaked in 5% benzophenone (BP) solution in *N*,*N*-dimethylformamide for 24 h, dried, quickly washed in ethanol and placed in a monomer mixture solution. UV-induced graft polymerization was performed using UV lamp OSRAM Ultra Vitalux E27 (UVA: 315–400 nm, 13.6 W; UVB: 280–315 nm, 3.0 W). It should be noted that UV-lamp heat the sample, without any cooling, the temperature increased to 85 °C. Air cooling allows to decrease the temperature to 35 °C. Finally, the samples were washed in water, dried at 50 °C, and weighed to determine the degree of grafting.

### 2.3. Membrane Characterization

FTIR analysis was performed using Agilent Cary 600 Series FTIR Spectrometer with attenuated total reflectance (ATR) accessory at scan range from 400 to 4000 cm^−1^, resolution 4.0 cm^−1^. X-ray photoelectron spectra were recorded on a Thermo Scientific K-Alpha spectrometer in the Ural Center for Shared Use “Modern Nanotechnology” Yekaterinburg, Russia. The pressure in the analysis chamber was maintained at 2 × 10^−6^ Pa or lower. Scanning electron microscope JEOL JSM-7500F was used for pore diameters measurements and morphology characterization. To estimate effective membrane pore sizes, the gas flow rate was used at a pressure drop of 20 kPa [34]. Colorimetric assay was done according to recommendations described in References [35,36].

### 2.4. Anodic Stripping Voltammetry Measurements

Sensors based on modified PET TeMs were obtained according to References [19,21] by magnetron sputtering of platinum with average thickness of 40–50 nm using mask on both sides of the membrane. These surfaces were connected to potentiostat EmStat 3+ (PalmSens) through 0.4 mm diameter copper cables, which were glued to metalized membrane using silver paste (Sigma-Aldrich, Seoul, South Korea). Connections were isolated by fingernail varnish and wax. Diameter of platinum surfaces is 5 mm. One side of membrane was used as working electrode, another side as counter electrode, Ag/AgCl electrode in 1 M KCl solution was used as reference electrode. Electrochemical analysis was performed by square wave anodic stripping voltammetry (SW-ASV) using standard solution of Cu^2+^, Pb^2+^, and Cd^2+^ in 0.1 M sodium acetate. Before the measurements, sensors were kept in analyzed solution for a certain time. Then applying −1.2 V for deposition time of 120 s and scanning from −1 V to 1 V at a frequency of 50 Hz and an amplitude of 20 mV, SW-ASV was performed.

## 3. Results and Discussion

### 3.1. UV-Induced Graft Polymerization of 4-vinylpyridine on PET TeMs

**Figure 1:**
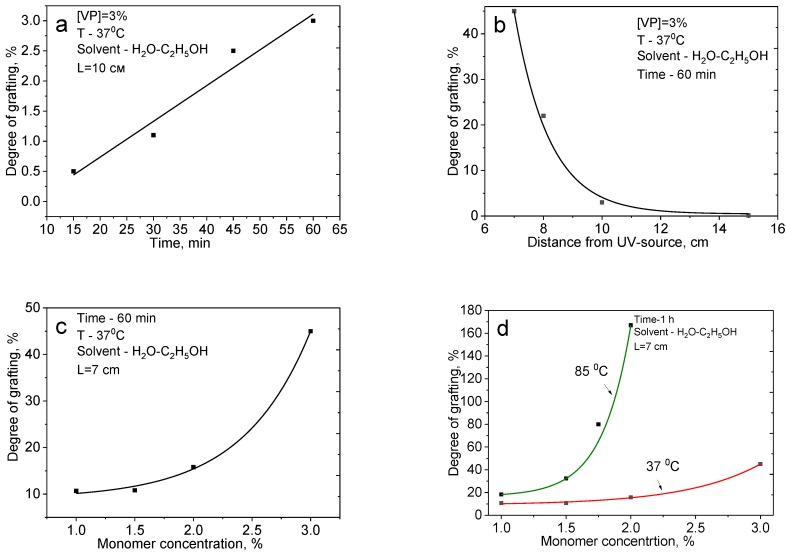
Grafting degree depends on irradiation time (**a**), distance to UV-source (**b**), monomer concentration (**c**), and temperature (**d**).

Optimization of irradiation time, distance from the UV-lamp and monomer concentration for the effective graft polymerization of 4-VPy on PET TeMs are presented in Figure 1. Water–ethanol mixture (30% by volume) was used as solvent. The choice of 30% water–ethanol mixture is due to the peculiarities of monomer dissolution. The solvent should dissolve the monomer well, at the same time it should not dissolve the previously adsorbed onto the membrane photosensitizer (benzophenone) in order to reduce its transition into the solution and reduce the side-reaction of homopolymerization.

As can be seen from Figure 1, there is an increase in the degree of grafting, measured gravimetrically with an increase in irradiation time, concentration, temperature, and a decrease in the distance from the UV source.

A sharp increase in the degree of grafting is observed (from 3 to 45%) with a decrease in the distance to the UV-source from 10 to 7 cm. An increase in temperature from 37 °C to 85 °C leads to an increase in the degree of grafting from 15% to 167% (at a concentration of monomer 2%, a distance of 7 cm). Such a sharp increase can lead to a significant change in the pore structure of the membranes.

**Figure 2:**
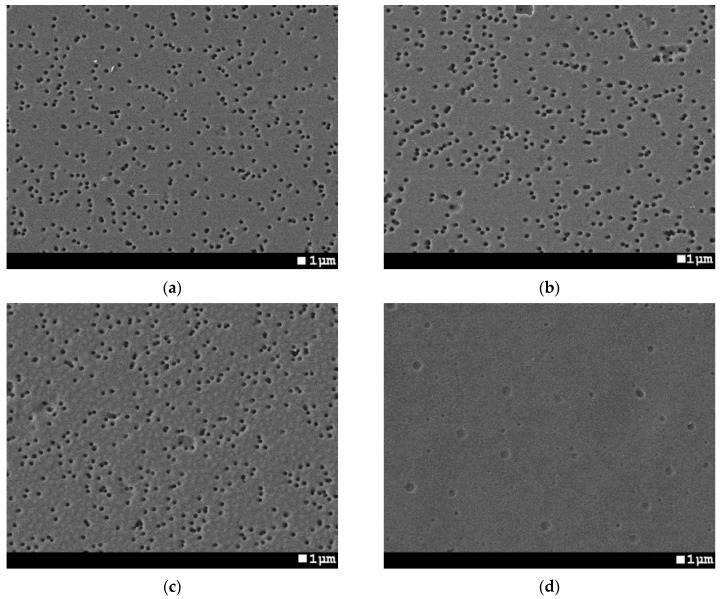
SEM images of poly(ethylene terephthalate) track-etched membranes (PET TeMs) before (**a**) and after UV-grafting of 4-vinylpyridine (4VPy) for 15 min (**b**), 60 min (**c**) (at constant T = 37°, L = 10 cm, monomer concertation 3%), and after grafting with grafting degree 167% (**d**).

At the same time, a prerequisite for the further use of such membranes as sensors is the preservation of the pore structure of the membranes; therefore, the samples were studied by scanning electron microscopy (SEM). Examples of SEM images with the preservation of the pore structure are presented in Figure 2. Data from SEM and gas permeability are summarized in Table 1.

As can be seen, with an increase in the degree of grafting, a uniform decrease in pore diameter occurs. According to gas permeability test, the pore diameter decreased from 400 nm to 355, 350, and 230 nm, respectively, at irradiation for 15, 30, and 60 min.

**Figure 3:**
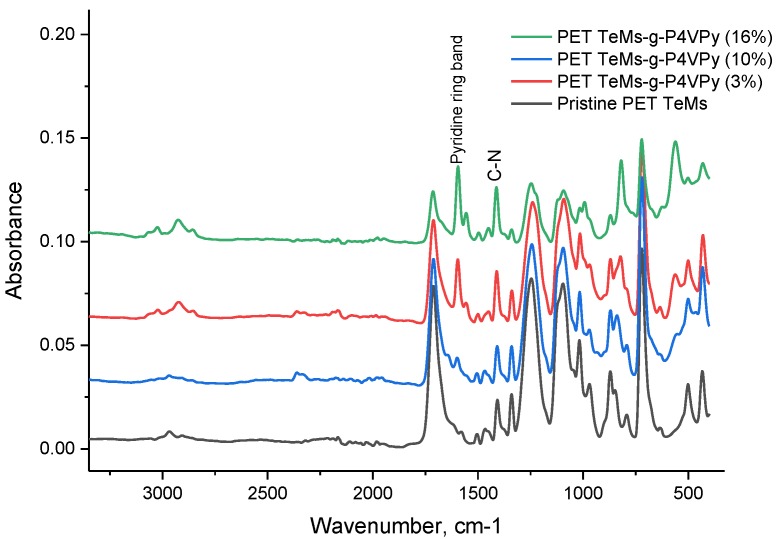
ATR-FTIR spectra of PET TeMs modified with 4-VPy at different grafting degrees.

Figure 3 shows FTIR-ATR spectroscopy that was used to elucidate changes on PET TeMs surface after 4-VPy grafting. The main absorption bands were at 3435 cm^−1^ (O–H), 2975 cm^−1^ (aromatic C–H), 2915 cm^−1^ (aliphatic CH), 1715 cm^−1^ (C=O), 1615, 1470, 1430, 1409 cm^−1^ (aromatic vibrations of the carbon skeleton), 1238 cm^−1^ (stretching vibrations of C (O)–O bonds), and 980 cm^−1^ (O–CH_2_) [37]. The main difference between the spectra of the initial and modified PET TeMs is related to P4VPy, absorption bands at 1595 cm^−1^ (C=C aryl.), 1450, and 1410 cm^−1^ (C–N). Moreover, an increase in the degree of grafting leads to increasing peak height at 1595 cm^−1^.

**Figure 4:**
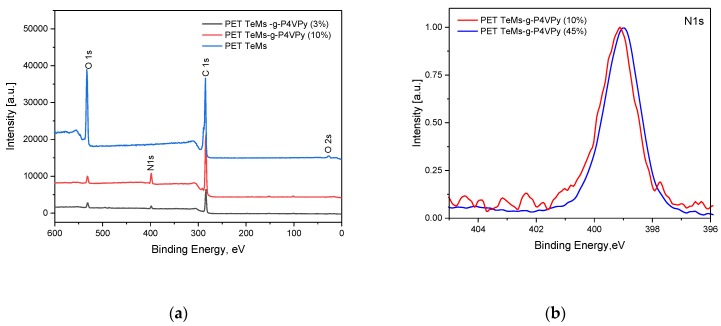

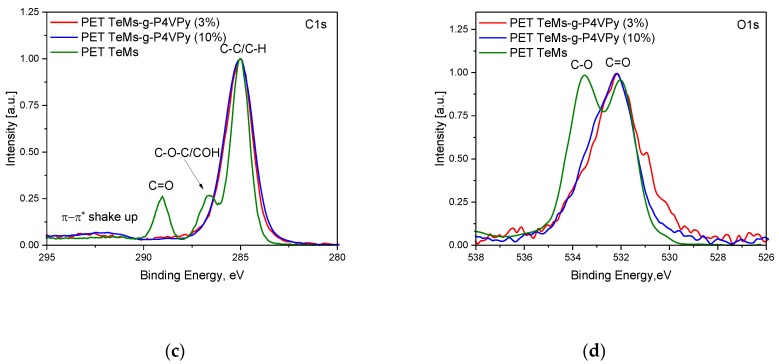
XPS survey (**a**), high resolution N1s (**b**), C1s (**c**), and O1s (**d**) spectra of PET TeMs before and after 4-VPy grafting at degrees of grafting of 3% and 10%.

X-ray photoelectron spectroscopy (XPS) analysis was carried out for further investigation of the surfaces of PET TeMs after grafting of 4-VPy with grafting degrees of 3% and 10%. Survey spectra of initial PET TeMs consist of carbon (71.9%) and oxygen (28.1%). Graft polymerization of 4-VPy at grafting degree of 3% lead to detection of 7% nitrogen, 7.8% of oxygen, and 85.2% of carbon. High-resolution N1s spectra of PET TeMs-g-P4VPy at different grafting degrees represents one peak at 399 eV related to nitrogen of P4VPy [38]. High-resolution C1s spectra of PET TeMs are consist of three main peaks at 285, 286.6, and 289 eV related to C–C/C–H, C–O–C, and C=O, respectively, also π–π* shake up was detected at 292 eV. Graft polymerization of P4VPy lead to disappearance of peaks at 286.6 and 289 eV, probably associated with full covering of very top surface with grafted polymer. This is also consistent with a decrease in intensities of C–O and C=O peaks in O1s spectra presented in Figure 4d.

### 3.2. UV-Induced Graft Copolymerization of 4-Vinylpyridine and Acrylic Acid on PET TeMs

**Figure 5:**
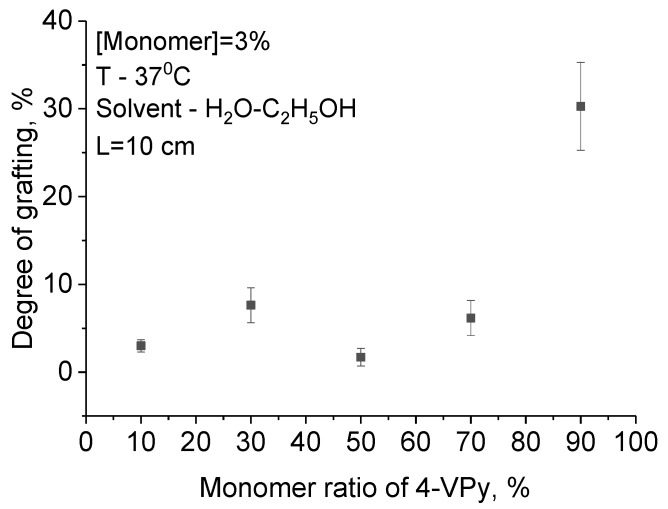
The dependence of the ratio of the monomers on the degree of grafting.

In order to study the effect of polymers of various nature on the detection ability, graft copolymerization of 4-VPy with AA was studied. AA contains negatively charged carboxyl groups (in contrast with positively charged P4VPy), which can also form complexes with heavy metal ions. Grafting polymerization was carried out in a water–ethanol medium by varying the concentration of monomers and the ratio of monomers, while the irradiation time and temperature remained constant—1 h and 37 °C, respectively. The results are presented in Figure 5.

**Figure 6:**
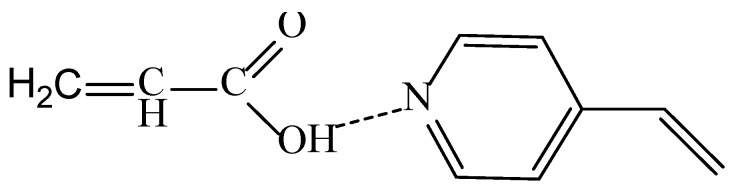
Interaction of acrylic acid and 4-vinylpyridine monomers.

It can be seen from the graph that the largest mass gain is observed at the ratio of the monomers of 4-VPy:AA = 90:10, probably due to the greater tendency of 4-VPy to polymerization compared to AA (r_1_ (4-VPy) = 5, r_2_ (AA) = 0.5 [39]. The minimum value of grafting degree is achieved when the ratio of monomers is 50:50. According to Reference [39], this is due to the formation of associates, which lead to a decrease in the reactivity of monomers (Figure 6).

**Figure 7:**
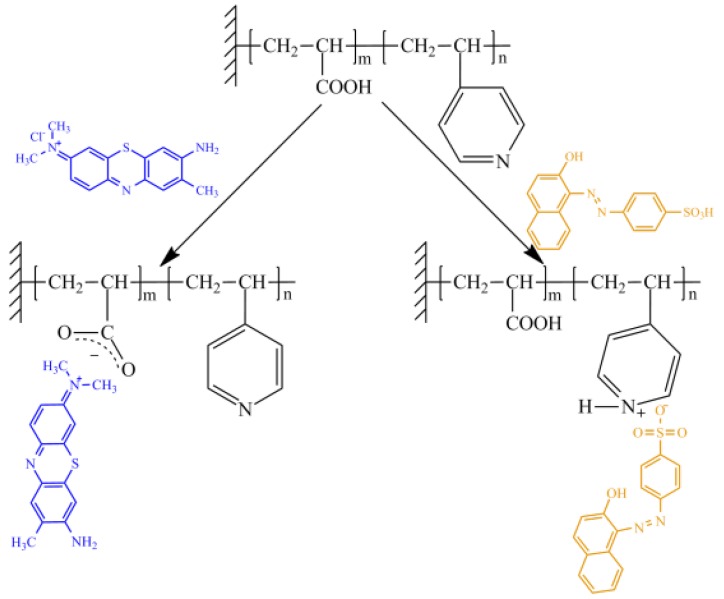
Schematic representation of the colorimetric analysis of the grafted copolymer on PET TeMs.

The composition of the grafted copolymer was studied by colorimetry using dyes toluidine blue (TB) and orange acid (AO) [35,36,40]. TB has specificity to carboxyl groups of PAA grafted chains and AO has specificity to P4VPy. Schematically, the process is presented in Figure 7. The results of the analysis are presented in Table 2.

As can be seen from the Table 2, the ratio of the polymer units in the chain 50:50 is achieved with the composition of the monomer mixture AA:4-VPy = 90:10, this is due to the high polymerization tendency of 4-VPy. However, with an increase in the concentration of 4-VPy, a decrease in its concentration in the grafted polymer is observed, probably due to the formation of a homopolymer, which was clearly recorded during the experiment. The observed effects are in good agreement with previously published works [41].

**Figure 8:**
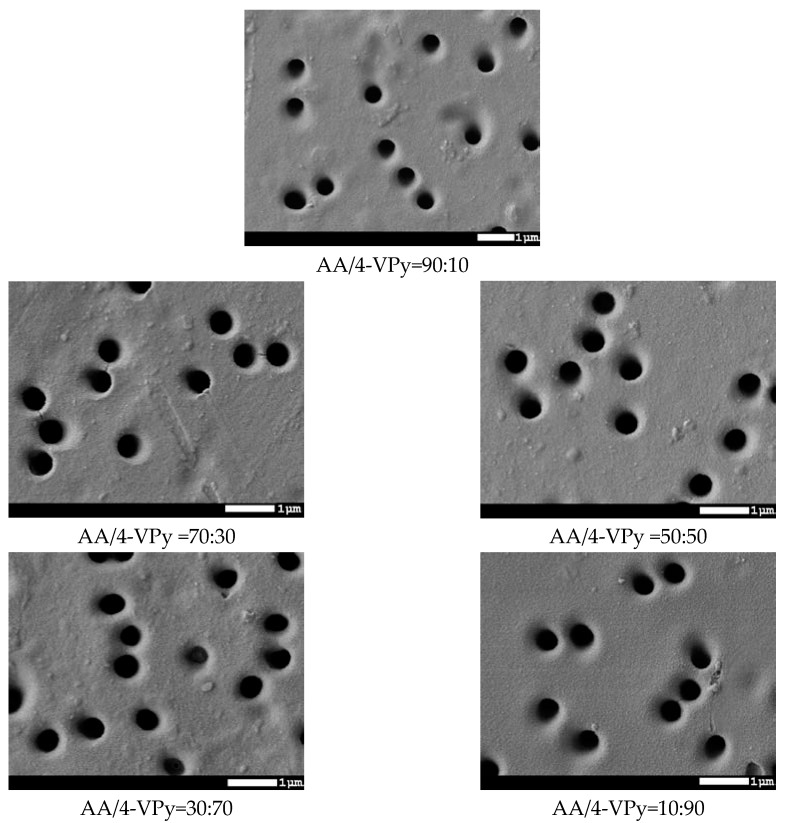
SEM images of PET TeMs modified with graft copolymerization of AA and 4VPy at different monomer ratios.

SEM images of the surface of modified PET TeMs at various monomer ratios are shown in Figure 8.

**Figure 9:**
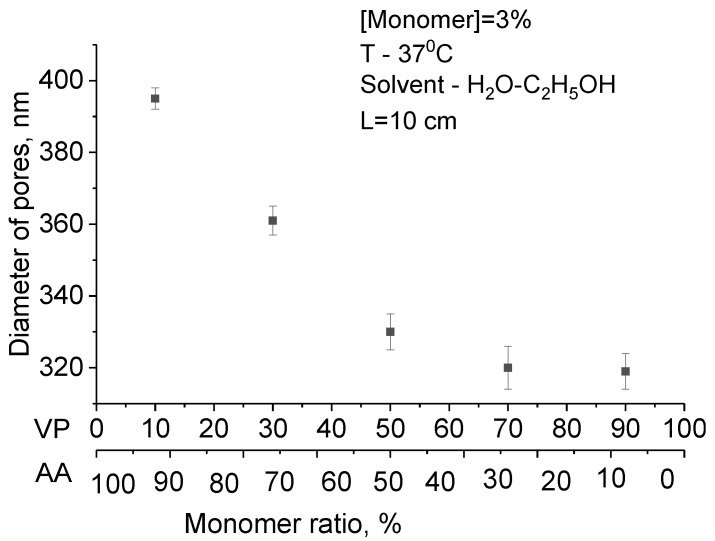
Pore diameters of PET TeMs at different monomer ratios.

SEM images clearly show a change in surface morphology. With increasing of grafting degree at monomer ratio AA/4-VPy = 30:70 and 10:90, surface topography inhomogeneity with inclusions can be observed. Moreover, changes in pore diameters occurred, and thickness of grafted copolymer is different between pore diameter of pristine PET TeMs and modified one. However, the pore diameter varies slightly. For a more accurate study of the change in pore diameter, the gas permeability method was used. The results are presented in Figure 9.

The gas permeability data obtained are in good agreement with SEM images. There is greater pore overgrowth with an increase in the proportion of 4-VPy in the monomer mixture.

To investigate chemical composition of the surface after graft polymerization, FTIR and XPS analysis were performed.

**Figure 10:**
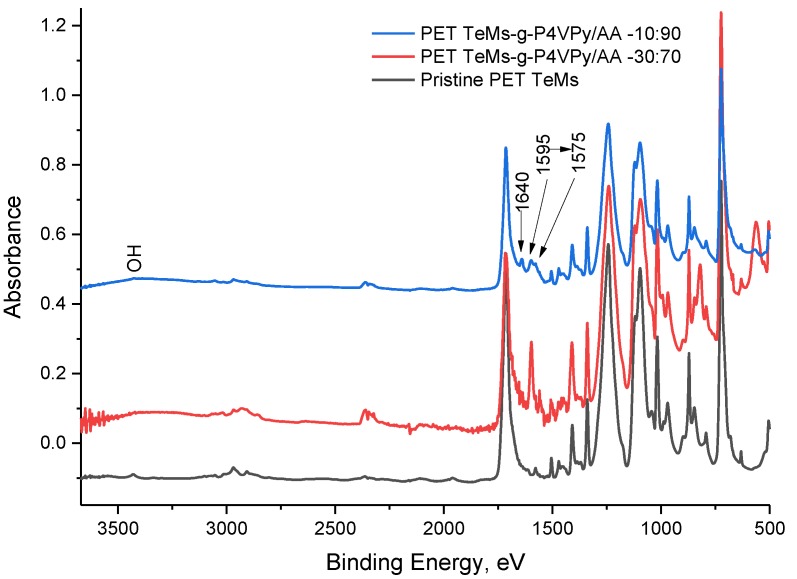
ATR-FTIR spectra of PET TeMs modified with 4-VPy/AA at different monomer ratios.

FTIR-ATR spectra are presented in Figure 10. In addition to the main peaks related to the PET membrane, shifting of C=C aryl of P4VPy ring from 1595 to 1575 cm^−1^ together with recording of peaks at 1640 cm^−1^ (COO^−^) and at 3500–3100 cm^−1^ (OH) was observed indicating formation complexing between PAA and P4VPy.

**Figure 11:**
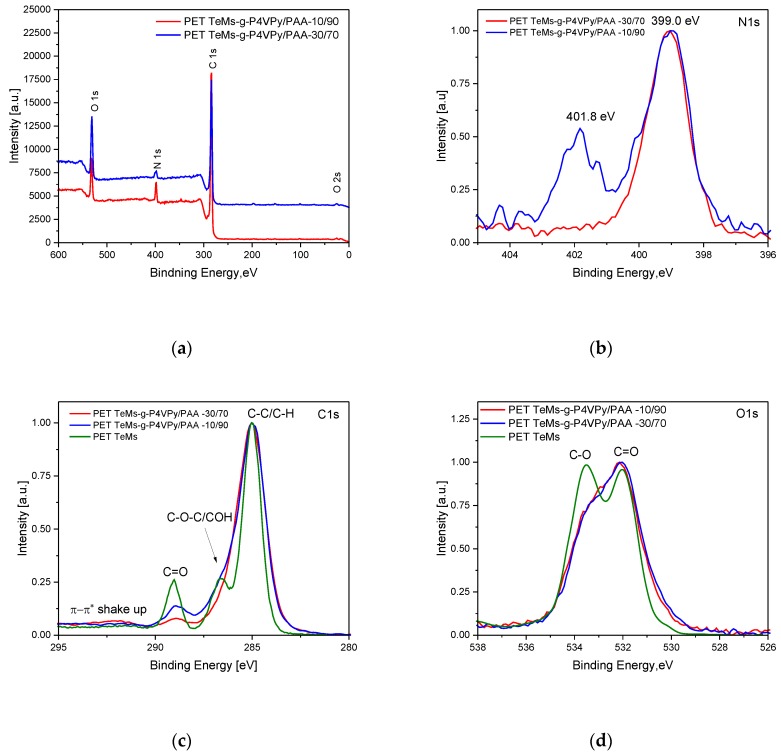
XPS survey (**a**), high-resolution N1s (**b**), C1s (**c**), and O1s (**d**) spectra of PET TeMs before and after graft copolymerization of 4-VPy and AA on PET TeMs at different monomer ratios.

Survey XPS spectra (Figure 11a) of PET TeMs-g-P4VPy/AA=30:70 consist of C (84.2%), O (9.3%), and N (6.5%); PET TeMs-g-P4VPy/AA = 10:90 consist of C (79.3%), O (16.4%), and N (4.3%). High-resolution C1s spectra show us that with increasing of AA content, an increase in the peak intensity at 289 eV corresponding to C=O bond has occurred. At a monomer mixture ratio of 4-VPy:AA = 10:90, which leads to the formation of copolymers on PET TeMs with a ratio of 50:50, two peaks appear at high-resolution N1s XPS spectra at 399 eV and 401.8 eV. The presence of a second peak at 401.8 eV also confirms the assumption that a complex is formed between PAA and P4VPy [38].

### 3.3. Electrochemical Detection

For the electrochemical detection, PET TeMs modified with AA according to our previously published paper [36] PET TeMs modified with 4-VPy and 4-VPy/AA in monomer ratio 10:90 (this monomer ration lead to formation 50:50 grafted PAA/P4VPy on the membrane, which in turn lead to the formation of a more stable complex with heavy metal ions [32]). In all cases the grafting degree was around 3%, in which the chemical modification of the surface was achieved by complexing groups to heavy metal ions (HMI) together with the preservation of the pore structure of the membranes. These modified membranes were converted to sensors by magnetron sputtering of platinum from both sides of the membranes with a thickness of 40–50 nm. One side of the membrane was used as working electrode, another side as counter electrode, thus distance between these two electrodes is equal to membrane thickness (12 µm) since graft polymerization at 3% of grafting degree does not lead to a significant change in membrane thickness.

**Figure 12:**
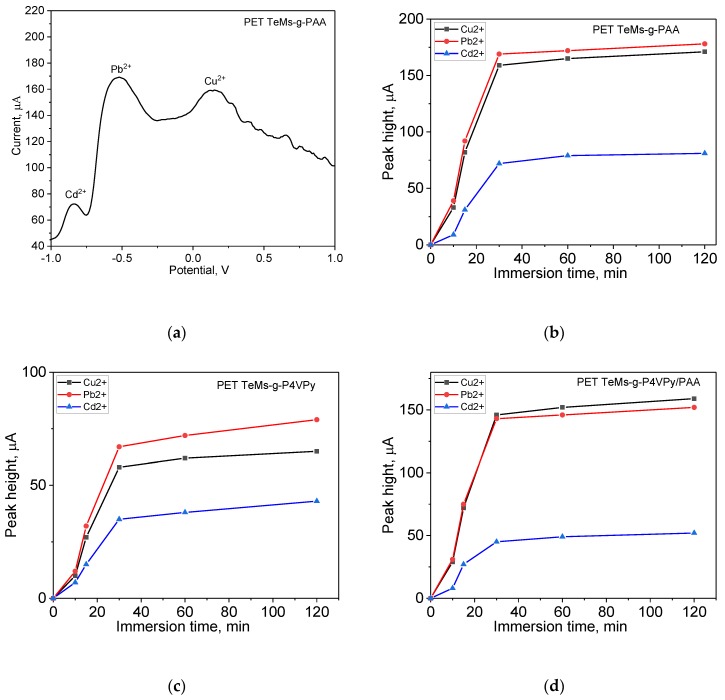
SW-ASV analysis curve after absorption time of 30 min (**a**) and kinetics of adsorption as function of HMI peak height (12.5 µg/L concentration), deposition at −1.2 V for 120 s (**b**–**d**).

**Figure 13:**
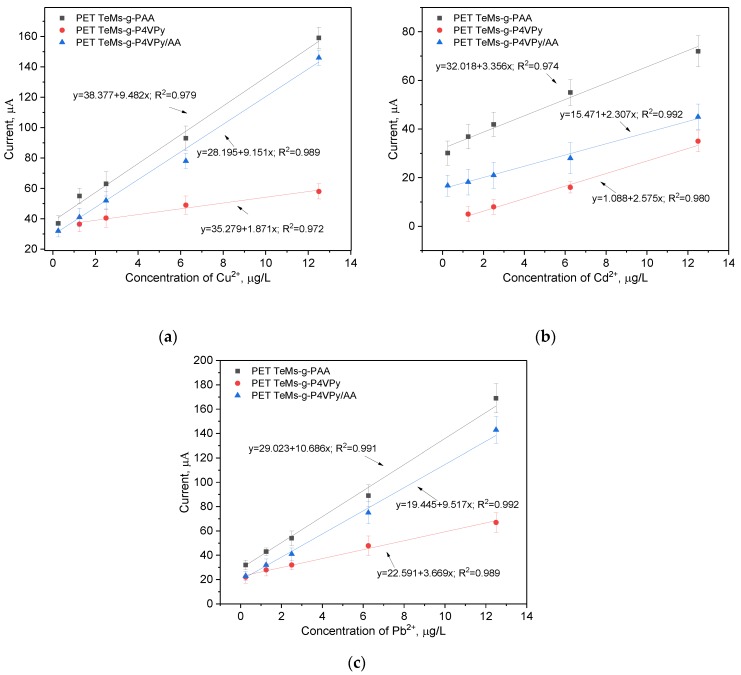
Calibration curves of peak currents for Cu^2+^ (**a**), Cd^2+^ (**b**), and Pb^2+^ (**c**) concentration for the SW-ASV after 30 min of adsorption in appropriate HMI solution in 0.1 M sodium acetate electrolyte.

SW-ASV was performed using standard solution of Cu^2+^, Pb^2+^, and Cd^2+^ in electrolyte of 0.1 M sodium acetate. HMIs were detected simultaneously in the same concentrations from 0.025 µg/L to 12 µg/L. For the optimization of adsorption time, sensors based on modified PET TeMs were immersed in 12.5 µg/L solution for certain time from 10 min to 120 min. Then, in present of reference electrode (Ag/AgCl, 1M KCl) at applied potential of −1.2 V during 120 s, deposition of HMI and their reduction was performed. After electrodeposition, a potential scan from −1 to 1 V was performed in order to oxidize of HMI at redox potential of Cd^2+^ around −0.84 V, Pb^2+^ at −0.52 V, and Cu^2+^ at 0.13 V, respectively. Results are presented in Figure 12. Optimal adsorption time for all HMI is 30 min. It should be noted that nonmodified membrane does not show any signal for HMI in this detection range. Then sensors were tested in different concentration, calibration curves are presented in Figure 13. The sensors modified with PAA and P4VPy/PAA were found to be more sensitive for copper and lead ions over cadmium ions, while sensors modified with P4VPy show less selectivity. Limits of detection (LOD) for sensors based on PET TeMs-g-PAA are 2.22, 1.05, and 2.53 µg/L for Cu^2+^, Pb^2+^, and Cd^2+^, respectively. LODs for sensors based on PET TeMs-g-P4VPy are 5.23 µg/L (Cu^2+^), 1.78 µg/L (Pb^2+^), and 3.64 µg/L (Cd^2+^) µg/L. PET TeMs-g-P4VPy/PAA electrodes are found to be sensitive with a LODs of 0.74 µg/L(Cu^2+^), 1.13 µg/L (Pb^2+^), and 2.07 µg/L(Cd^2+^). In most cases, good correlation between concentration and current was found and R^2^ is achieved to 0.992. Thus, these sensors can be used for SW-ASV detection of copper, lead and cadmium in the µg/L range.

Furthermore, it is seen that LODs of cadmium ion is higher than LODs of copper and lead ions, this fact is in a good correlation with complexing ability of this ion with polymers: cadmium reacts weaker with polymers than lead and copper [42,43].

## 4. Conclusions

This work demonstrates the methods of modification of track-etched membranes based on poly(ethylene terephthalate) by methods of UV-induced graft polymerization of 4-vinylpyridine, acrylic acid and copolymerization of 4-vinylpyridine and acrylic acid for application as detectors for electrochemical detection of heavy metal ions using square-wave anodic stripping voltammetry. Optimal conditions of modification were found leading to creation of anchors for heavy metal ions complexation and prevention of pore structure of the membranes. Based on such modified membranes, detectors were prepared by platinum sputtering through the mask on both sides of the membranes. These sensors can be used for detection of copper, lead, and cadmium ions in the concentration range from 0.25 to 12.5 µg/L. Membranes modified with acrylic acid and acrylic acid/4-vinylpyridine showed more selectivity to Pb^2+^ and Cu^2+^ rather than to Cd^2+^. Modification by copolymerization with different polymer nature (polyacrylic acid and poly-4-vinylpyridine) leads to more accurate simultaneous heavy metal ions detection.

## Figures and Tables

**Table 1:** Pore sizes for grafted PET TeMs at various conditions.

№ Sample	Time of Grafting, min	Concentration of Monomer,%	Distance from UV-Lamp, cm	Temperature, °C	Grafting Degree, %	Effective Pore Size, nm	Pore Size (from SEM Analysis), nm	Porosity, %
1	0	-	-	-	-	400 ± 5	405 ± 25	22
2	15	3	10	37	0.5	355 ± 4	365 ± 17	17
3	30	3	10	37	1	350 ± 6	361 ± 15	16.5
4	60	3	10	37	3	230 ± 6	253 ± 22	7
5	60	3	7	37	45	0	0	-
6	60	3	15	37	0.1	357 ± 6	376 ± 22	17
7	60	1	7	37	10	145 ± 4	167 ± 21	4
8	60	1.5	7	37	11	154 ± 6	176 ± 15	4
9	60	2	7	37	16	110 ± 5	103 ± 12	2
10	60	3	7	37	45	0	0	-
11	60	1	7	85	18	0	0	-
12	60	1.5	7	85	32	0	0	-
13	60	2	7	85	167	0	0	-

**Table 2:** The composition of the grafted copolymer PET-TeMs-g-PAA/poly(4-vinylpyridine) (P4VPy) depending on the AA/4VPy ratio.

Monomer Mixture Composition		Dye Concentration, µM/g		Composition of Grafted Copolymer	
[AA]	[4-VPy]	TB	AO	m_1_	m_2_
90	10	11.9	11.1	51.7	48.3
70	30	21.4	89.5	19.3	80.7
50	50	26.6	72.1	27.0	73.0
30	70	25.7	81.9	23.9	76.1
10	90	34.0	44.4	43.4	56.6

## References

[1] Kumar P., Kim K.H., Bansal V., Lazarides T., Kumar N. (2017). Progress in the sensing techniques for heavy metal ions using nanomaterials. J. Ind. Eng. Chem..

[2] Cui L., Wu J., Ju H. (2015). Electrochemical sensing of heavy metal ions with inorganic, organic and bio-materials. Biosens. Bioelectron..

[3] (2011). Guidelines for Drinking-Water Quality.

[4] Pierce D.T., Zhao J.X. (2015). Trace Analysis with Nanomaterials.

[5] Dai X., Wu S., Li S. (2018). Progress on electrochemical sensors for the determination of heavy metal ions from contaminated water. J. Chin. Adv. Mater. Soc..

[6] Lu Y., Liang X., Niyungeko C., Zhou J., Xu J., Tian G. (2018). A review of the identification and detection of heavy metal ions in the environment by voltammetry. Talanta.

[7] Abdulla M., Ali A., Jamal R., Bakri T., Wu W., Abdiryim T. (2019). Electrochemical sensor of double-thiol linked PProDOT@Si composite for simultaneous detection of Cd(II), Pb(II), and Hg(II). Polymers.

[8] Brett C.M.A., Brett A.M.O. (1993). Electrochemistry: Principles, Methods, and Applications.

[9] Huang H., Chen T., Liu X., Ma H. (2014). Ultrasensitive and simultaneous detection of heavy metal ions based on three-dimensional graphene-carbon nanotubes hybrid electrode materials. Anal. Chim. Acta.

[10] Gao C., Yu X.-Y., Xu R.-X., Liu J.-H., Huang X.-J. (2012). AlOOH-Reduced Graphene Oxide Nanocomposites: One-Pot Hydrothermal Synthesis and Their Enhanced Electrochemical Activity for Heavy Metal Ions. ACS Appl. Mater. Interfaces.

[11] Liu X., Yao Y., Ying Y., Ping J. (2019). Recent advances in nanomaterial-enabled screen-printed electrochemical sensors for heavy metal detection. TrAC Trends Anal. Chem..

[12] Zhao W., Ge P.-Y., Xu J.-J., Chen H.-Y. (2007). Catalytic Deposition of Pb on Regenerated Gold Nanofilm Surface and Its Application in Selective Determination of Pb ^2+^. Langmuir.

[13] Ouyang R., Bragg S.A., Chambers J.Q., Xue Z.-L. (2012). Flower-like self-assembly of gold nanoparticles for highly sensitive electrochemical detection of chromium(VI). Anal. Chim. Acta.

[14] Wang N., Kanhere E., Miao J., Triantafyllou M.S. (2018). Nanoparticles-modified chemical sensor fabricated on a flexible polymer substrate for cadmium(II) detection. Polymers.

[15] Gouveia-Caridade C., Brett C.M.A. (2008). Strategies, Development and Applications of Polymer-Modified Electrodes for Stripping Analysis. Curr. Anal. Chem..

[16] Kim Y., Amemiya S. (2008). Stripping Analysis of Nanomolar Perchlorate in Drinking Water with a Voltammetric Ion-Selective Electrode Based on Thin-Layer Liquid Membrane. Anal. Chem..

[17] Abdalla N.S., Amr A.E.-G.E., El-Tantawy A.S.M., Al-Omar M.A., Kamel A.H., Khalifa N.M. (2019). Tailor-Made Specific Recognition of Cyromazine Pesticide Integrated in a Potentiometric Strip Cell for Environmental and Food Analysis. Polymers.

[18] Pinaeva U., Dietz T.C., Al Sheikhly M., Balanzat E., Castellino M., Wade T.L., Clochard M.C. (2019). Bis[2-(methacryloyloxy)ethyl] phosphate radiografted into track-etched PVDF for uranium (VI) determination by means of cathodic stripping voltammetry. React. Funct. Polym..

[19] Bessbousse H., Zran N., Fauléau J., Godin B., Lemée V., Wade T., Clochard M.-C. (2016). Poly(4-vinyl pyridine) radiografted PVDF track etched membranes as sensors for monitoring trace mercury in water. Radiat. Phys. Chem..

[20] Barsbay M., Güven O., Bessbousse H., Wade T.L., Beuneu F., Clochard M.-C. (2013). Nanopore size tuning of polymeric membranes using the RAFT-mediated radical polymerization. J. Membr. Sci..

[21] Bessbousse H., Nandhakumar I., Decker M., Barsbay M., Cuscito O., Lairez D., Clochard M.-C., Wade T.L. (2011). Functionalized nanoporous track-etched β-PVDF membrane electrodes for lead(ii) determination by square wave anodic stripping voltammetry. Anal. Methods.

[22] Mizuguchi H., Shibuya K., Fuse A., Hamada T., Iiyama M., Tachibana K., Nishina T., Shida J. (2012). A dual-electrode flow sensor fabricated using track-etched microporous membranes. Talanta.

[23] Mizuguchi H., Sakurai J., Kinoshita Y., Iiyama M., Kijima T., Tachibana K., Nishina T., Shida J. (2013). Flow-based Biosensing System for Glucose Fabricated by Using Track-etched Microporous Membrane Electrodes. Chem. Lett..

[24] Apel P.Y., Blonskaya I.V., Dmitriev S.N., Orelovich O.L., Sartowska B.A. (2015). Ion track symmetric and asymmetric nanopores in polyethylene terephthalate foils for versatile applications. Nucl. Instrum. Methods Phys. Res. Sect. B Beam Interact. Mater. Atoms.

[25] Apel P.Y. (2019). Fabrication of functional micro- and nanoporous materials from polymers modified by swift heavy ions. Radiat. Phys. Chem..

[26] Pinaeva U., Lairez D., Oral O., Faber A., Clochard M.-C., Wade T.L., Moreau P., Ghestem J.-P., Vivier M., Ammor S. (2019). Early Warning Sensors for Monitoring Mercury in Water. J. Hazard. Mater..

[27] Ashraf S., Cluley A., Mercado C., Mueller A. (2011). Imprinted polymers for the removal of heavy metal ions from water. Water Sci. Technol..

[28] Zhao G., Huang X., Tang Z., Huang Q., Niu F., Wang X. (2018). Polymer-based nanocomposites for heavy metal ions removal from aqueous solution: A review. Polym. Chem..

[29] Zhang Y., Vallin J.R., Sahoo J.K., Gao F., Boudouris B.W., Webber M.J., Phillip W.A. (2018). High-Affinity Detection and Capture of Heavy Metal Contaminants using Block Polymer Composite Membranes. ACS Cent. Sci..

[30] Oyagi M.O., Onyatta J.O., Kamau G.N., Guto P.M. (2016). Validation of the polyacrylic acid/glassy carbon differential pulse anodic stripping voltammetric sensor for simultaneous analysis of lead(II), cadmium(II) and cobalt(II) ions. Int. J. Electrochem. Sci..

[31] Ling J.L.W., Ab Ghani S. (2013). Poly(4-vinylpyridine-co-aniline)-modified electrode—Synthesis, characterization, and application as cadmium(II) ion sensor. J. Solid State Electrochem..

[32] Kabanov N.M., Kozhevnikova N.A., Kokorin A.I., Rogacheva V.B., Zezin A.B., Kabanov V.A. (1979). Study of the structure of a polyacrylic acid-Cu (II)-poly-4-vinylpyridine ternary polymeric metal complex. Polym. Sci. U.S.S.R..

[33] Turmanova S., Vassilev K., Boneva S. (2008). Preparation, structure and properties of metal-copolymer complexes of poly-4-vinylpyridine radiation-grafted onto polymer films. React. Funct. Polym..

[34] Mulder M. (1996). Transport in Membranes. Basic Principles of Membrane Technology.

[35] Korolkov I.V., Mashentseva A.A., Güven O., Gorin Y.G., Zdorovets M.V. (2018). Protein fouling of modified microporous PET track-etched membranes. Radiat. Phys. Chem..

[36] Korolkov I.V., Mashentseva A.A., Güven O., Zdorovets M.V. (2017). Modification of Track-Etched PET Membranes by Graft Copolymerization of Acrylic Acid and N-Vinylimidazole. Pet. Chem..

[37] Holland B., Hay J. (2002). The thermal degradation of PET and analogous polyesters measured by thermal analysis–Fourier transform infrared spectroscopy. Polymer.

[38] Zhou X., Goh S.H., Lee S.Y., Tan K.L. (1998). XPS and FTi.r. studies of interactions in poly(carboxylic acid)/poly(vinylpyridine) complexes. Polymer.

[39] Fujimori K., Trainor G.T., Costigan M.J. (1984). Complexation and rate of polymerization of acrylic acid and methacrylic acid in the presence of poly(4-vinylpyridine) in dilute methanol solution. J. Polym. Sci. Polym. Chem. Ed..

[40] Hennig A., Borcherding H., Jaeger C., Hatami S., Würth C., Hoffmann A., Hoffmann K., Thiele T., Schedler U., Resch-Genger U. (2012). Scope and Limitations of Surface Functional Group Quantification Methods: Exploratory Study with Poly(acrylic acid)-Grafted Micro- and Nanoparticles. J. Am. Chem. Soc..

[41] Masuda S., Minagawa K., Tsuda M., Tanaka M. (2001). Spontaneous copolymerization of acrylic acid with 4-vinylpyridine and microscopic acid dissociation of the alternating copolymer. Eur. Polym. J..

[42] Moulay S., Bensacia N. (2016). Removal of heavy metals by homolytically functionalized poly(acrylic acid) with hydroquinone. Int. J. Ind. Chem..

[43] Biswas T.K., Yusoff M.M., Sarjadi M.S., Arshad S.E., Musta B., Rahman M.L. (2019). Ion-imprinted polymer for selective separation of cobalt, cadmium and lead ions from aqueous media. Sep. Sci. Technol..

